# Randomized clinical trial comparing removal followed by topical imiquimod *versus* removal followed by topical methylprednisolone in the treatment of keloids

**DOI:** 10.1590/acb406225

**Published:** 2025-08-25

**Authors:** Alexandre Spiandorello Ricciardi, Marcio Fernandes Chedid, Claudia Elizabeth Thompson, Rafaela Katrine da Silva, David Rubem Azulay, Mônica Manela-Azulay

**Affiliations:** 1 Hospital Nossa Senhora da Conceição – Plastic Surgery Service – Porto Alegre (RS) – Brazil.; 2 Universidade Federal do Rio Grande do Sul – Postgraduate Program in Medicine: Surgical Sciences – Porto Alegre (RS) – Brazil.; 3 Hospital de Clínicas de Porto Alegre – Digestive System Surgery Service – Porto Alegre (RS) – Brazil.; 4 Universidade Federal de Ciências da Saúde de Porto Alegre – Department of Pharmacosciences – Graduate Program in Health Sciences – Porto Alegre (RS) – Brazil.; 5 Universidade Federal do Rio de Janeiro – Postgraduate Program in Dermatology – Rio de Janeiro (RJ) – Brazil.

**Keywords:** Keloid, Imiquimod, Steroids, Methylprednisolone, Clinical Trial

## Abstract

**Purpose:**

Keloids are unaesthetic benign dermatosis characterized by a disorganized proliferation of collagen. Treatment of keloids constitutes a therapeutic challenge. The aim of this study was to evaluate the efficacy and effectiveness of topical imiquimod associated with surgical excision in the treatment of keloid.

**Methods:**

A randomized, double blind, matching-lesion (self-paired manner) clinical trial. Ten patients with two keloid lesions each in similar anatomical and contralateral areas (paired lesions) had their keloids excised, and the operative site treated with the application of 5% imiquimod cream or 0.1% methylprednisolone aceponate cream (gold standard) for eight weeks.

**Results:**

Eight patients (total = 16 lesions) completed the study. Four of the total eight keloids (50%) in the methylprednisolone group vs. 3/8 keloids (37.5%) in the imiquimod group recurred in the first post-treatment year (*p* 0.05).

**Conclusion:**

Surgical removal plus application of topical imiquimod was shown as safe, and its efficacy was not statistically inferior for the treatment of keloids as compared to methylprednisolone. Due to the lack of efficacy in most therapeutic modalities, surgical removal plus topical imiquimod could be recommended as an additional first line therapy and especially for recurrent keloids. Studies with larger samples are necessary to evaluatre therapies for keloids.

## Introduction

Keloids are abnormal erythematous fibrous growths that rise above the level of the skin. They can develop after stimuli such as cutaneous surgeries for the treatment of skin cancer. Keloids also may rise following burns, trauma, lacerations, acne, piercing placement, although there is a description of spontaneous occurrence. The epidemiology of keloids varies widely among populations, with an incidence of 4.5–16% in black and Hispanic people^1^. In a case-control study in Cameroon, the prevalence found in the population was 3.5%^2^.

The excess of cellular matrix with the increase in the number of fibroblast mitoses and their resistance to cellular apoptosis causes an increased mesenchymal response, which contributes to the formation of keloids. The deep inflammatory lesion in the reticular dermis that characterizes the hallmark of the keloid is the hyalinized thickened collagen, although it is not always possible to detect it, which can make it difficult to distinguish it from the hypertrophic scar^3^. Brieflly, it is possible to differentiate keloids from hypertrophic scars considering some characteristics related to different anatomical sites and sizes, for example. In keloids, growth is expected beyond the external limits of the original wound, while in hypertrophic scars it tends to respect the limits. The keloid has a disordered size, not respecting the limits of thickness and verticality, whereas the hypertrophic scar is rarely larger than 1 cm. The hypertrophic scar does not have any specific anatomical site of occurrence. However, the keloid usually appears on the chest, back, shoulders, nape, and earlobe, for example. The hypertrophic scar tends to regress spontaneously, but the same behavior is not found in the case of keloids.

Imiquimod is an imidazoquinolinamine that has the property of modifying innate and acquired immunity through binding to Toll-like receptors, which induce numerous cytokines in cells such as keratinocytes, CD4 lymphocytes, cytotoxic T lymphocytes, macrophages, NK cells, Langherhans cells, monocytes, and fibroblasts. The induction of tumor necrosis factor alpha, interleukin-12, interferon alpha and interferon gamma lead to local inflammatory phenomena. Imiquimod reduces collagen and glycosaminoglycan synthesis and induces apoptosis, in addition to being able to alter the p53 gene expression in fibroblast apoptosis^4^.

There are a few prior studies on keloid removal plus imiquimode for the treatment of keloids, and most of them consist of small case series^5^–^10^. So far, only two prior randomized clinical trials (RCTs) evaluated the efficacy of surgery plus imiquimod^11^,^12^. None of the two RCTs has compared surgery plus imiquimod to the gold standard treatment in the literature: keloid removal plus radiotherapy or local injection of steroids (triamcynolone)^13^.

The aim of this RCT was to evaluate the efficacy and effectiveness of topical imiquimod associated with surgery in the treatment of keloids. The outcomes of keloids treated with surgery plus application of topical imiquimod were compared to those of keloids treated with surgical excision plus application of topical methylprednisolone. The recurrence of keloids one year after treatment was the main outcome. The presence and the degree of symptoms (pain, itching), as well as the degree of patient’s satisfaction (directly linked to the recurrence criterion) after one year of treatment, were also evaluated.

## Methods

The study was a randomized (simple randomization blocked), double-blind, matching-lesion self-paired type design. Patient recruitment was based on referral by medical colleagues. The places of reception for the patients were the dermatological surgery outpatient clinics of the Dermatology Services of the Clementino Fraga Filho University Hospital of Rio de Janeiro (HUCFF-RJ), the Professor Rubem David Azulay Dermatology Institute of the Santa Casa de Misericórdia of Rio de Janeiro (IDPRDA-SCMRJ) and the Bonsucesso General Hospital of Rio de Janeiro (HGB-RJ). The preoperative evaluation was carried out after filling out a questionnaire and ordering the following preoperative tests: hemoglobin levels, hematocrit, bleeding time, coagulation time, urea, creatinine, and fasting glucose.

Patients were informed of the risks and benefits of the treatment and signed an informed consent form. The project, including the informed consent form, was approved by the Research Ethics Committee (CEP) of the University Hospital Clementino Fraga Filho (CEP-HUCFF 198/03), and surgical procedures and research were performed at the same hospital.

**Figure 1:**
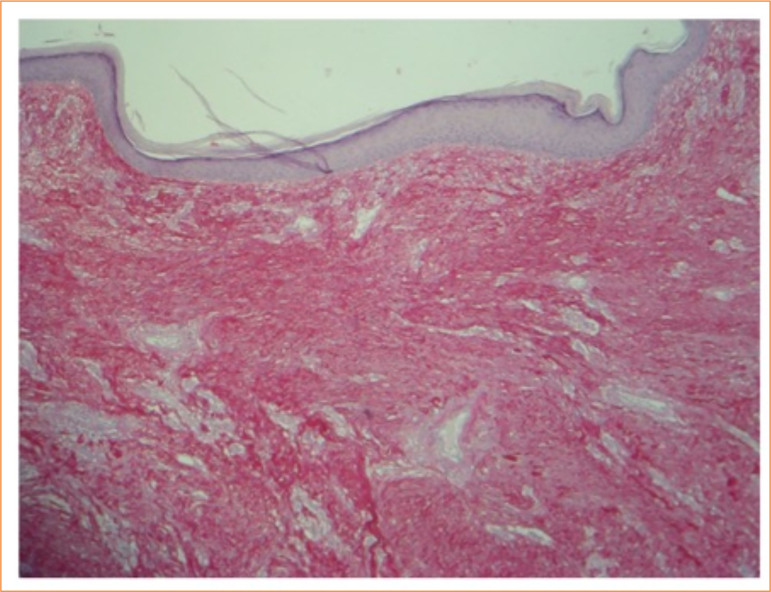
Keloid histology (picrosirius red staining).

Source: Elaborated by the authors.

The total study sample included 10 patients who had at least two keloid lesions, totaling 20 keloid lesions (Fig. 1). There were eight patients with outer ear keloids (a total of 16 keloids), one patient with upper limb keloids (a total of two keloids), and one patient with chest keloids (a total of two keloids). Therefore, the anatomical areas were similar and contralateral, configuring paired lesions. In the sample, there were phototypes III, IV, V and VI.

The keloids were excised, and the operative site was treated with the application of topical substances. After the clinical diagnosis and the excision of the entire keloid lesion, the material was fixed in formaldehyde at 10% and included in paraffin, following the techniques conventional processing.

All patients in the sample received surgical treatment and started applying 5% imiquimod cream the night after the procedure in only one of the keloid lesions (one lesion per patient). The use of imiquimod cream continued the night shift for a period of eight weeks.

The control group was the excised contralateral lesions, paired by anatomical site, which received surgical and topical treatment, with the application of 0.1% methylprednisolone aceponate cream starting in the night following the procedure and maintaining up daily use for a period of eight weeks.

Topical drugs provided in the study were stored in unidentified tubes with the capacity of 15 grams each. The patients and the dermatological surgeon did not know which of the two topical drugs was being used in which lesion, during the eight-week period (time of drug treatment).

In addition to the evaluation of the material stained by hematoxylin-eosin, histochemistry techniques were performed in Alcian-blue, ph = 2.5 for detection of glycosaminoglycans with digestion by streptococcal hyaluronidase, and of hyaluronic acid.

Histochemistry was performed on Alcian-blue, ph = 1.0, for detection of dermatan–sulfate, heparan-sulfate, and chondroitin-sulfate, and histochemistry in acid periodic Schiff (PAS) for detection of interactive glycoproteins (fibronectin) with digestion by amylase. It was further performed histochemistry with Red from Picrosirius to characterization of both collagens type I and type III in keloids.

### Inclusion criteria

The study included patients between 18 and 70 years old with phototypes from III to VI, followed at the dermatological surgery outpatient clinics of the Dermatology Services of the HUCFF-RJ, IDPRDA-SCMRJ and HGB-RJ. Patients should have at least one pair of keloid lesions in any part of the body, for at least six months of existence (clinical diagnosis), of variable dimensions, with diameters within the open range of 0.5 to 5 cm, and no previous treatment in the last two months.

### Exclusion criteria

Pregnant and/or breastfeeding patients, patients using oral retinoids or who had suspended their use for a period of less than one year, patients with pre-existing severe heart disease, uncontrolled hypertension, diabetics, nephropathies were excluded from the study. Malnourished, obese (body mass index > 30 kg/m^2^), smokers, alcoholics, patients with autoimmune diseases and/or with blood dyscrasias, or immunosuppression or immunodepression, women using oral hormonal contraceptives and patients with keloids in the occipital/posterior cervical and presternal region were also excluded from this study.

### Clinical-surgical evaluation

Keloids ranged in diameter between 0.5 and 5 cm. After surgery (at the end of one year of evaluation), it was expected to find a normotrophic, linear scar, with no recurrence. Scar was clinically defined by the presence of a postoperative indurated papule or nodule that remained in place for the follow-up period and that extended beyond the scar limits. The recurrence criterion was measured through the observation of two evaluators (including the researcher), who answered the questionnaire on the presence or absence of the lesion in question. Measurements of keloid dimensions, thickness, length, and width were performed, with comparison after the end of the study. Dimensional measurements were confirmed both at the time of macroscopic anatomopathological analysis.

The presence or absence of pain in the lesion and pruritus was evaluated before and after treatment (questionnaire answered by the patients), as well as the presence or absence of induration and keloid color change before and after treatment (questionnaire answered by the doctor).

Surgical revision consultations took place on the seventh, 14th, and 21st postoperative day, according to the previous location of the keloid area and to assess possible complications such as local infection, suture dehiscence, and hematoma. The therapeutic response and prevention of possible side effects of the drugs used such as effectiveness, erythema, pain, itching, erosion, atrophy, and hyperpigmentation of the applied sites were evaluated. Follow-up included periodic review visits at four weeks after the procedure, and after eight, 12, 16, 20, 24, 36 and 48 weeks.

### Surgical technique

Antisepsis was performed with topical and degerming povidine, surgical drapes, local peri and infralesional infiltrative anesthesia with a 1- or 3-mL syringe, coupled to the insulin needle, according to the solution prepared with 0.9%–10 mL saline, lidocaine a 2%–5 mL approximately 0.6% in the total dilution, NaHCO3 8.4%–2 mL, and epinephrine 1:1,000-0.2 mL, approximately 1:200,000 in the total dilution. Surgical incision, removal, and manipulation of the surgical lesion and packaging of the specimen were performed in a 10% aqueous formalin flask. Revision of hemostasis, detachment of the surgical plane, interrupted simple suture (square knot), with monofilament thread in a single plane, occurred. Correlations was done between operated anatomical areas and adequate use of monofilament threads according to the Table 1.

**Table 1:** Demographic and clinicopathological characteristics of ten patients included in the study.

Characteristics	(n = 10)
Age, mean, Years old	21.9 ± 4.8, range 18–31
Duration of lesion in years, mean	2.1 ± 0.5
Sex (female) (%)	62.50
Frequency of keloids according to phototype	
III (%)	12.50
IV (%)	12.50
V (%)	37.50
VI (%)	37.50
Frequency of keloids according to precipitating factor	
Ear ring (%)	75
Local trauma (%)	12,50
Spontaneous (%)	12.50

Source: Elaborated by the authors.

### Postoperative

The postoperative site was covered with a hypoallergenic microporous tape, and patients were instructed to start topical drug therapy in each lesion in the immediate postoperative period. The return for the first surgical revision occurred in seven days with removal of the suture, according to anatomical location. In the ear lobes, the suture was removed 14 days after the excision, and in the upper limbs and chest after 21 days. The antibiotics used in the postoperative period were azithromycin 500 mg for three days, and the prescribed analgesia was acetaminophen 500 mg q6h.

### Histopathological evaluation

**Figure 2:**
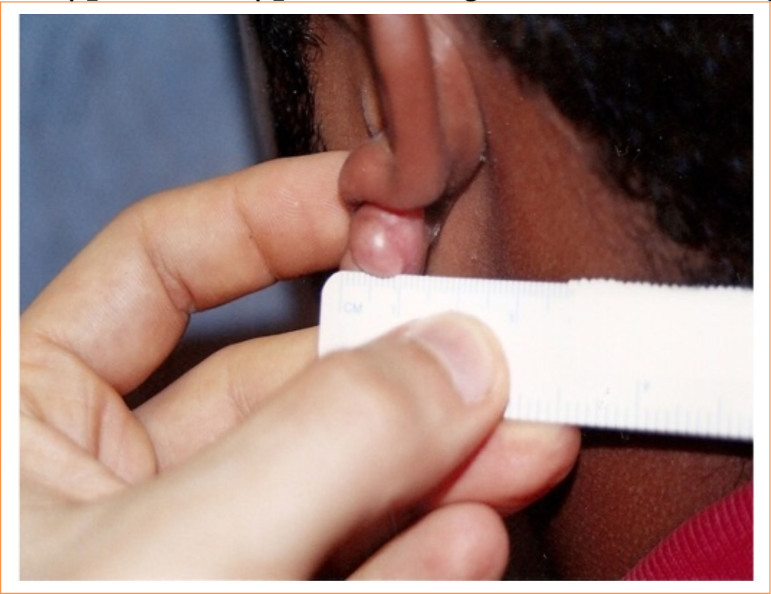
Right ear keloid.

Source: Elaborated by the authors.

For histological evaluation, the material was fixed in 10% formalin and embedded in paraffin, following the conventional processing techniques for hematoxylin-eosin. Alcian-blue pH 2.5 was used to detect glycosaminoglycans with digestion by streptococcal hyaluronidase and hyaluronic acid. Alcian-blue, pH 1.0 was used for detection of dermatan-sulfate, heparan-sulfate, and chondroitin-sulfate PAS for detection of interacting glycoproteins (fibronectin) with amylase digestion. Picrosirius red was used for characterization of both type I and type III collagen in keloids (Fig. 2).

### Psychological assessment

For the summary psychological assessment, the degree of patient satisfaction with the established therapy was measured on a scale from 0 to 1–score 0 equals to dissatisfied, and score 1 equals to satisfied.

### Statistical analysis

The sample size was calculated using the formula for the difference observed between two proportions. As mentioned above, the unit was lesion (two in each patient), as that the groups would be of the same sizes (matching-lesion), and the same patient serves as control.

Similarly to mentioned above, it was expected that the proportion of improvement in one of treatments was 20% (p 1 = 0.20) (group of lesions treated surgically + topical application of methylprednisolone) and that in other treatment was 80% (p 2 = 0.80) (group of lesions treated surgically + application topical of imiquimod). For an α error = 5%, and a β error = 10% (power of the study 90%), the sample size would be 20 lesions.

For statistical evaluation (analysis), paired t-test–comparison between the various continuous variables, McNemar test (non-parametric test)–ideal for analyzing two dichotomous variables (proportions) in self-paired studies were employed. McNemar uses the χ^2^ distribution. For both cases, any result with a chance of error lower than 5% (*p* < 0.05) was considered significant. All data were entered into Excel and later exported to the Statistical Package for the Social Sciences (SPSS version 11.0).

## Results

A total of 10 patients was evaluated in this double-blind RCT. Each patient had two lesions (a total of 20 lesions), being eight patients with bilateral external ear keloids, one patient with upper limb keloids, and one patient with chest keloids. The mean patient age was 21.9 ± 4.80, ranging between 18 and 31 years old, and 62.5% of patients were female. The mean duration of the lesions was 2.1 ± 0.5 years. The distribution of frequency of keloids according to phototypes III, IV, V, and VI was 12.5, 12.5, 37.5, and 37.5%, respectively. The frequency of keloids according to the precipitating factor was 75% for ear rings, 12.5% for local traumas, and 12,5% for spontaneous factor (Table 1).

**Figure 3:**
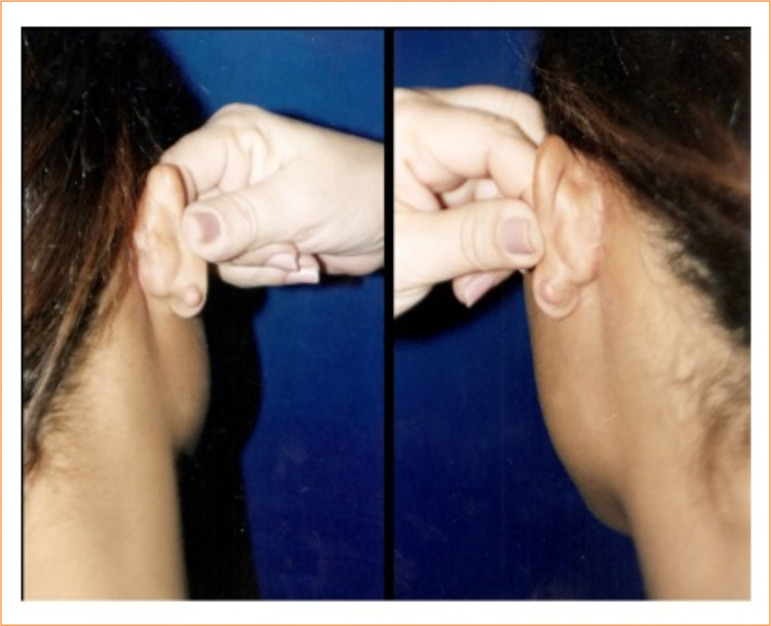
Bilateral year keloid (before excision).

Source: Elaborated by the authors.

**Figure 4:**
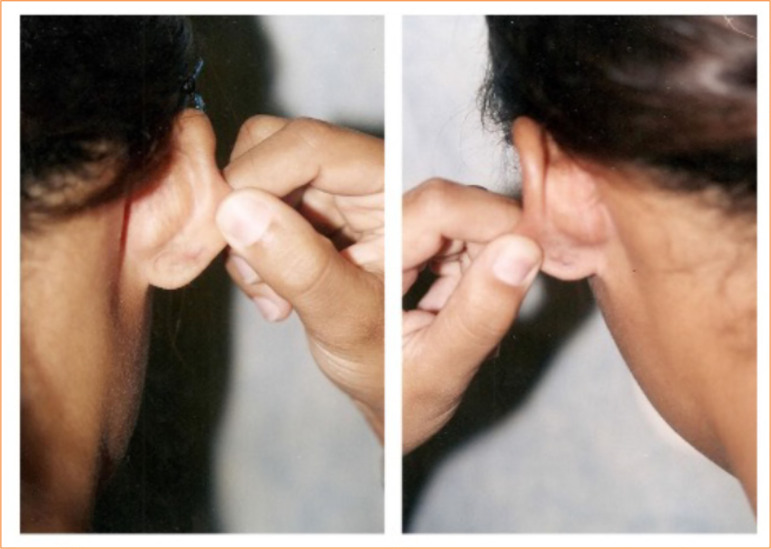
Ears of the same patient after bilateral ear keloid.

Source: Elaborated by the authors.

Eight patients (in a total of 16 lesions, eight of them in the imiquimod group vs. eight in the steroid group) completed the study: six patients with bilateral lesions in the external ear, one patient with lesions in the chest, and one patient with bilateral lesions in the upper limbs. All 10 patients concluded the treatment (Figs. 3 and 4). However, two of them did not return to the one-year visits after treatment. They both were contacted by phone, being alive and without any serious side effects. As they did not attend personally, they were not examined by the medical team, and, consequently, it was not possible to evaluate the recurrence of keloids.

No side effects were observed in the methylprednisolone patient group. However, statistically significant side effects occurred in the imiquimod group: erythema in 6/8 patients vs. zero in the steroid group (*p* < 0.01 in comparison to the methylprednisolone group); erosion in five patients vs. zero in the steroid group (*p* < 0.007 in comparison to the methylprednisolone group), and pain in four patients vs. zero in the steroid group (*p* < 0.02 in comparison to the methylprednisolone group).

About the symptomatology of patients (eight who remained in the study, therefore 16 lesions), after one year of treatment with the drugs involved, we observed varied complaints of itching, pain in three patients (in the six lesions paired and treated with different medications). In one patient, there were symptoms only in one of the two treated lesions, from a total of 16 lesions; in this case, the area treated with methylprednisolone. All side effects disappeared with cessation of the treatment.

Regarding the presence of symptoms one year after treatment, complaints of itching and pain were present in three patients (in both of their paired lesions treated with different topical cream). In one patient, there were symptoms in only one of the two lesions treated (in this case, with methylprednisolone). Thus, symptoms were present in 4/8 patients in the methylprednisolone group (50%) vs. 3/8 patients in the imiquimod group (*p* > 0.05).

Four of the total eight keloids (50%) treated with surgery plus methylprednisolone vs. 3/8 keloids (37.5%) treatment with surgery plus imiquimod recurred in the first post-treatment year (*p* > 0.05). The interobserver agreement for all 16 lesions analyzed one year after treatment (Kappa’s test) was 100%. Finally, in relation to the degree of therapeutic satisfaction after one year, we did not observe statistically significant differences among treatments, since in five lesions treated with imiquimod and four treated with methylprednisolone patients were satisfied (*p* > 0.05).

## Discussion

This is the first RCT evaluating the efficacy of surgery plus imiquimod in the treatment of keloids. In this double-blind RCT, the efficacy and effectiveness of keloid removal followed by topical application of 5% imiquimod cream were compared to the efficacy and effectiveness of keloid removal followed by topical application of methylpredinisolone. Moreover, it was the only RCT comparing the efficacy of surgery plus imiquimod to that of surgery plus steroids (in this case, methylpredinsolone). Another strength of this study is the fact it is a paired study. As a consequence, the individual biases related to the analysis of symptoms and patients’ satisfaction were minimized.

Only two prior RCTs evaluated the efficacy of surgery plus imiquimod up to date^11^,^12^. The first one evaluating surgical removal plus imiquimod topical application versus surgery removal only included a total of five patients^11^. In that case, the recurrence of keloids was three out of the five patients (60% recurrence) treated in both groups. The second RCT was the one of Berman et al.^12^. The authors included a total of 10 patients whose keloid scars were surgically removed, with the patients randomly divided into two groups and instructed to apply 5% imiquimod daily versus vehicle cream. Eight of the total 10 patients completed the six months of the study. The local recurrence rate of keloids was 37.5% in the imiquimod group vs. 75% in the control group (*p* = 0.54)^12^.

In the present study, one year after keloid removal plus starting topical imiquimod therapy, the treatment was effective in 62.5% of cases (five lesions of eight lesions treated out). The recurrence rate of the lesions treated with surgery plus imiquimod was 37.5% (three of eight lesions), whereas the recurrence rate of lesions treated with removal plus 0.1% methylprednisolone cream was 50% (four lesions of eight lesions). There was no statistical significance between the two groups.

Including both RCTs^11^,^12^ and few non-RCT studies on surgery plus imiquimod for keloids^5^–^10^, the recurrence rate for surgery plus imiquimod ranged from 3^10^ to 91%^5^. The causes for this heterogeneity in results can be likely attributed, at least in part, to the site of the keloid. The lowest recurrence rate (3%) was reported in the study performed in ear keloids in Thailand by Chuangsuwanich and Gunjittisomrarn^10^, whereas the highest recurrence rate (91%) was reported in the study by Cacao et al.^5^ involving chest keloids.

Regarding the presence of symptoms one year after treatment, symptoms were present in 4/8 patients in the methylprednisolone group (50%) *vs*. 3/8 patients in the imiquimod group (37.5%). Interestingly, three of those four patients had symptoms in the lesion treated with surgery plus imiquimod and also in the lesion treated with surgery plus methylprednisolone. The studies reported in the literature did not evaluate the presence of symptoms one year after the treatment, which reinforce the importance of the follow-up performed in our study.

The degree of satisfaction in our study was analyzed for each lesion treated. There was satisfaction in five lesions treated with imiquimod without recurrence, while four patients with no recurrence of the keloid were satisfied with the methylprednisolone treatment. Thus, the patient satisfaction was closely related to therapeutic success, and there is no additional study in the literature evaluating the presence of symptoms one year after the treatment.

The difference of 12.5% in recurrence (37.5% for imiquimod group *vs*. 50% for methylpredinisolone group) was not statistically significant. However, two out of 10 patients did not show up for clinical evaluation (both patients were alive and without any serious side effects). Thus, a total of 16 lesions was evaluated in final follow-up. So, the present RCT did not reach the total of 20 lesions, being underpowered to discard the occurrence of a type-II error. Another potential limitation was the fact that methylprednisolone rather than injection of tramcinolone was employed after keloid removal. The authors wanted the drugs to be administered by the same route, so that the study would be double-blind, and thus results would be more comparable. Topical methylprednisolone has chemical characteristics and moderate potency, similar to those of triamcinolone (gold standard for keloid treatment)^14^. Both medications decrease the synthesis of pro-collagen, glycosaminoglycans, and fibronectin. Thus, methylprednisolone and triamcinolone posess anti-inflammatory, anti-proliferative and anti-pruritic effects. Moreover, highly lipophilic agents, such as MPA, generally do not attain high serum concentrations, thus reducing thepotential for systemic side effects^15^.

## Conclusion

Surgical removal plus application of topical imiquimod was shown as safe and with an efficacy not statistically lower than surgery plus topical methylprednisolone. Imiquimod, however, led to more local side effects than methylprednisolone. Due to the lack of efficacy in most therapeutic modalities for this case, surgical removal plus topical imiquimod could be recommended as an additional therapy for keloids, especially to those refractories to surgery plus steroid therapy. Studies with larger samples and follow-up are necessary to look for additional therapies for keloids.

## Data Availability

The data will be available upon request.
